# Supplementary material for: Dysfunctional TRPM8 signalling in the vascular response to environmental cold in ageing
Source: eLife. 2021 Nov 2;10:e70153. doi: 10.7554/eLife.70153 (PMC8592571; doi:10.7554/eLife.70153)
Supplement: Supplementary file 1. [file elife-70153-supp1.docx]

**Supplementary Table 1.** List of primer sequences

| **Gene Name** | **RefSeq ID** | **Forward Sequence** | **Reverse Sequence** | **Amplicon Size (bp)** |
| --- | --- | --- | --- | --- |
| ***Trpa1*** | NM_001348288.1 | GAGGATTGCTATGCAGGTGGA | TCCACTTTGCGCAAGTACCA | 75 |
| ***Trpv1*** | NM_001001445.2 | CAACAAGAAGGGGCTTACACC | TCTGGAGAATGTAGGCCAAGAC | 77 |
| ***Trpm8*** | NM_134252.4 | TTGTATTCCGGCTCCACTCTTC | AGTTCCTGCTGACGGTGAAAA | 120 |
| ***α_2A_*** | NM_007417.5 | TCATCTCCTCGTCCATCGGT | ACGCTTGGCGATCTGGTAAA | 86 |
| ***α_2c_*** | NM_007418.3 | ACAAGCGCACTCTCCAATCA | AGTCTCCACCTCACTCGGTT | 106 |
| ***P16*** | NM_001040654.1 | CCATCTGGAGCAGCATGGAGT | TCATCATCACCTGAATCGGGGTA | 150 |
| ***P21*** | NM_001111099.2 | CAGCAGAATAAAAGGTGCCACA | CACGGGACCGAAGAGACAAC | 100 |
| ***Gapdh*** | NM_001289726.1 | GGTCATCCCAGAGCTGAACG | TTGCTGTTGAAGTCGCAGGA | 294 |
| ***B2m*** | NM_009735.3 | GCCTGTATGCTATCCAGAAAACCCC | TGTGAGGCGGGTGGAACTGTG | 114 |
| ***Act*** | NM_007393.5 | CACTGTCGAGTCGCGTCCA | GTCATCCATGGCGAACTGGTG | 90 |
| ***Hprt*** | NM_013556.2 | TCCTCCTCAGACCGCTTTT | CCTGGTTCATCATCGCTAATC | 90 |
| ***Dbh*** | NM_138942.3 | TACTTTGCGGATGCCTGGAG | ATCTCGAGTCCTCTGTGCCT | 93 |
| ***Th*** | NM_009377.2 | AGGGCCTCTATGCTACCCAT | AAGCCAGTCCGTTCCTTCAA | 136 |

Act- beta actin

HPRT- hypoxanthine guanine phosphoribosyl transferase

B2M - β2 microglobulin

GAPDH - Glyceraldehyde 3-phosphate dehydrogenase

Dbh – dopamine β-hydroxylase

TH – tyrosine hydroxylase

α_2a_– alpha2a adrenoceptor

α_2c_– alpha2c adrenoceptor
