# Supplementary material for: Dysfunctional TRPM8 signalling in the vascular response to environmental cold in ageing
Source: eLife. 2021 Nov 2;10:e70153. doi: 10.7554/eLife.70153 (PMC8592571; doi:10.7554/eLife.70153)
Supplement: Source data 1. [file elife-70153-supp2.docx]

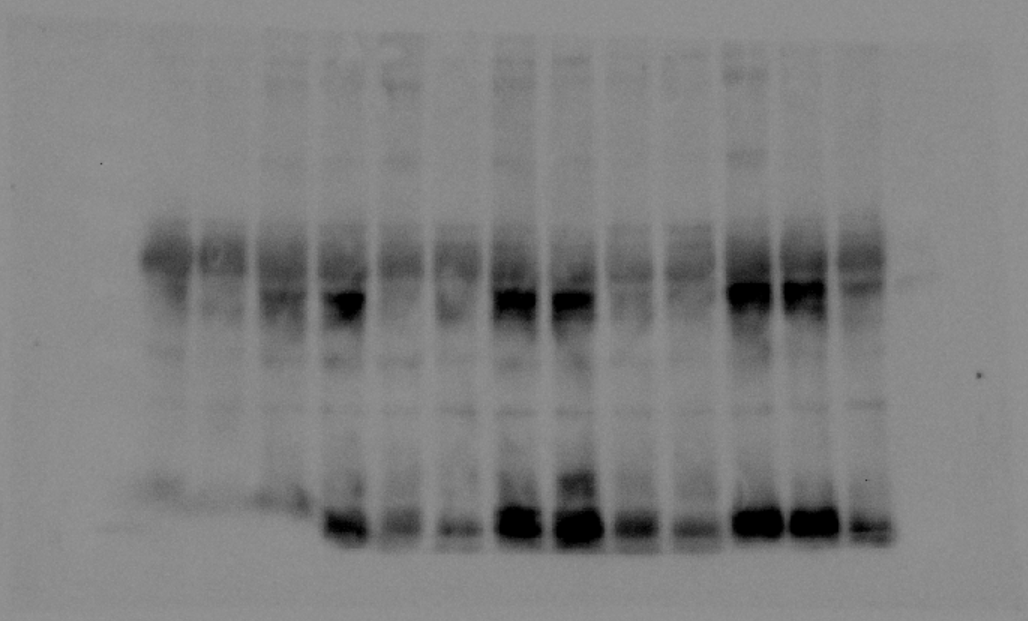

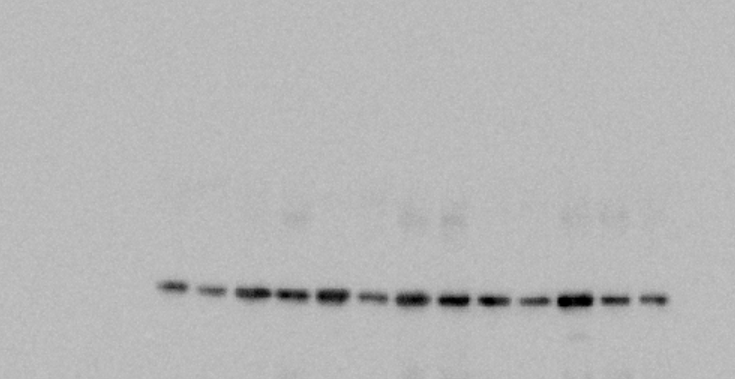


**P21**

**GAPDH**

**21kDa**

**37kDa**

Figure 1(i): Original uncropped blot of p21 and GAPDH. The yellow box represents the cropped region used for the manuscript.


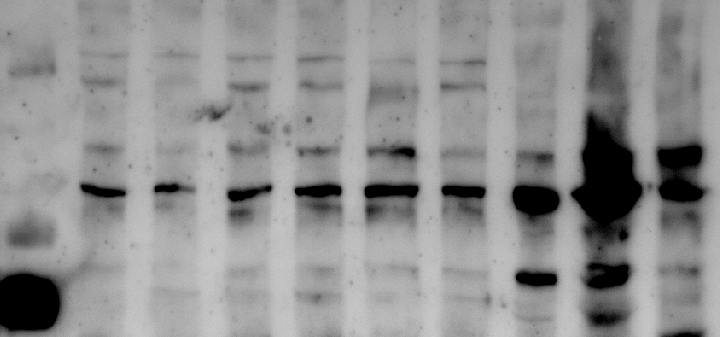

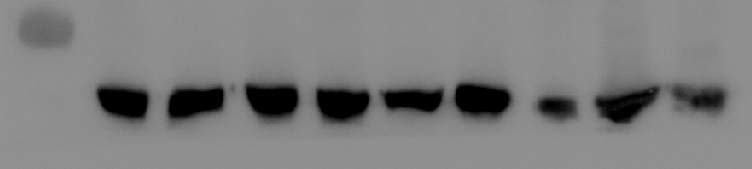


**TRPM8**

**Tubulin**

**130kDa**

**100kDa**

**70kDa**

**70kDa**

Figure 4(i): Original uncropped blot of TRPM8 and Tubulin. The yellow box represents the cropped region used for the manuscript.


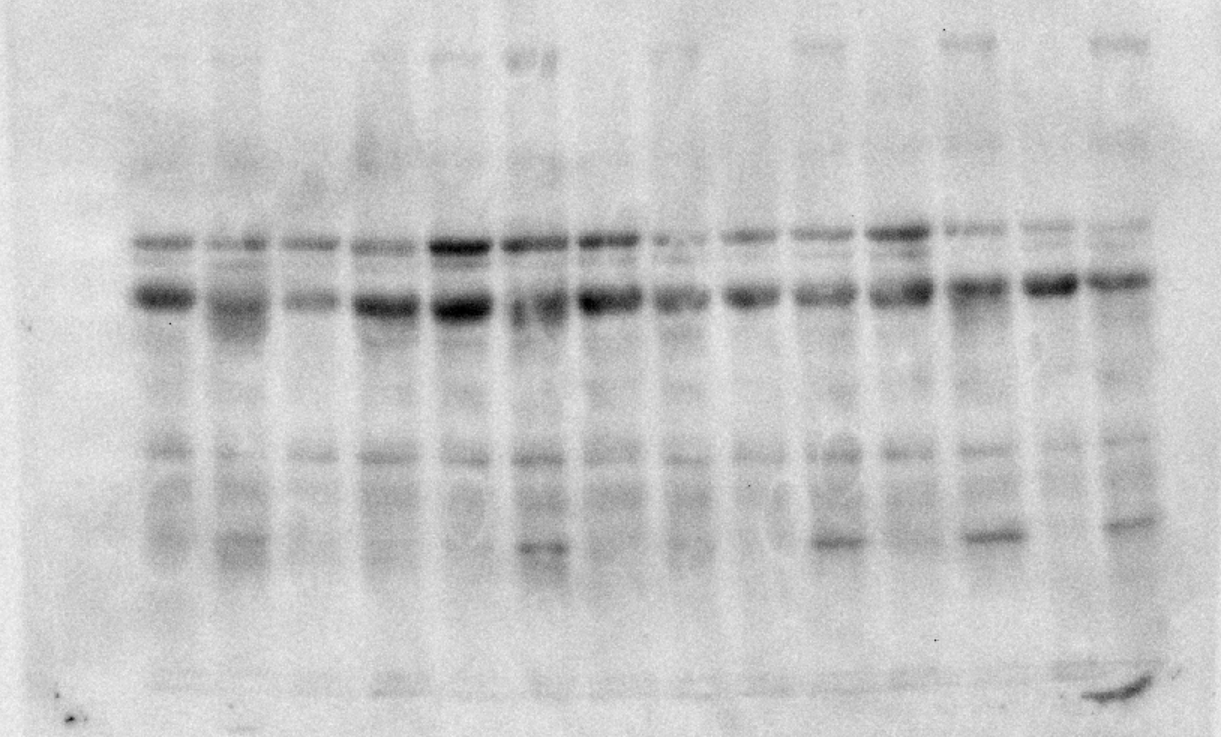

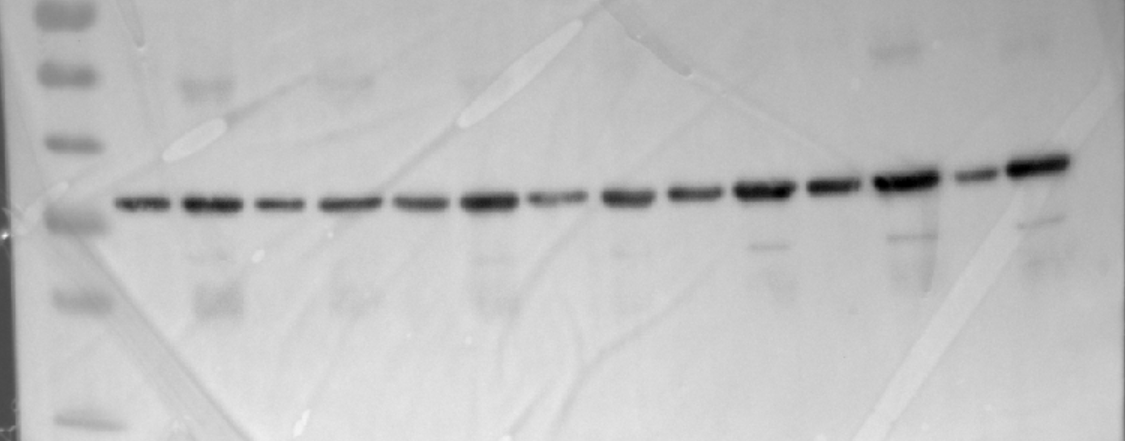


**Alpha2c**

**GAPDH**

**35kDa**

**~55kDa**

Figure 6(e): Original uncropped blot of alpha2c and GAPDH. The yellow box represents the cropped region used for the manuscript.


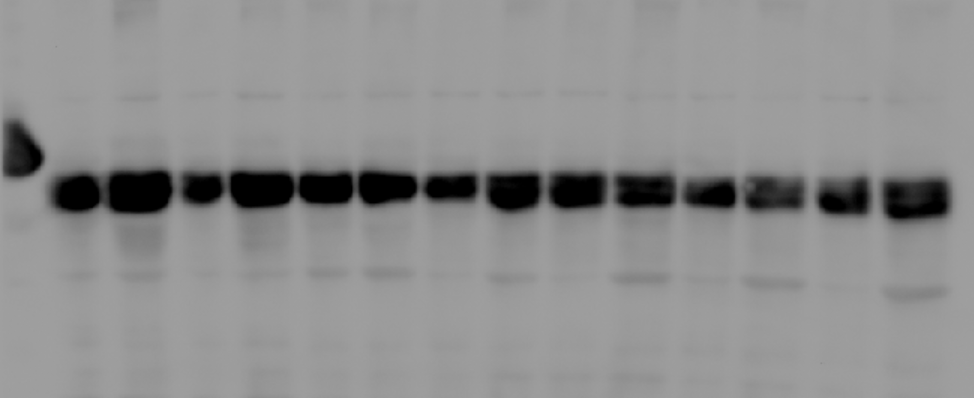

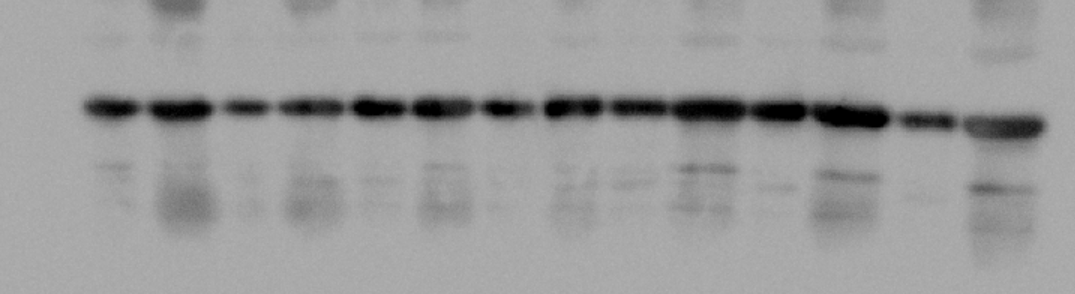


**TH**

**GAPDH**

**70kDa**

**37kDa**

Figure 6(f): Original uncropped blot of tyrosine hydroxylase (TH) and GAPDH. The yellow box represents the cropped region used for the manuscript.


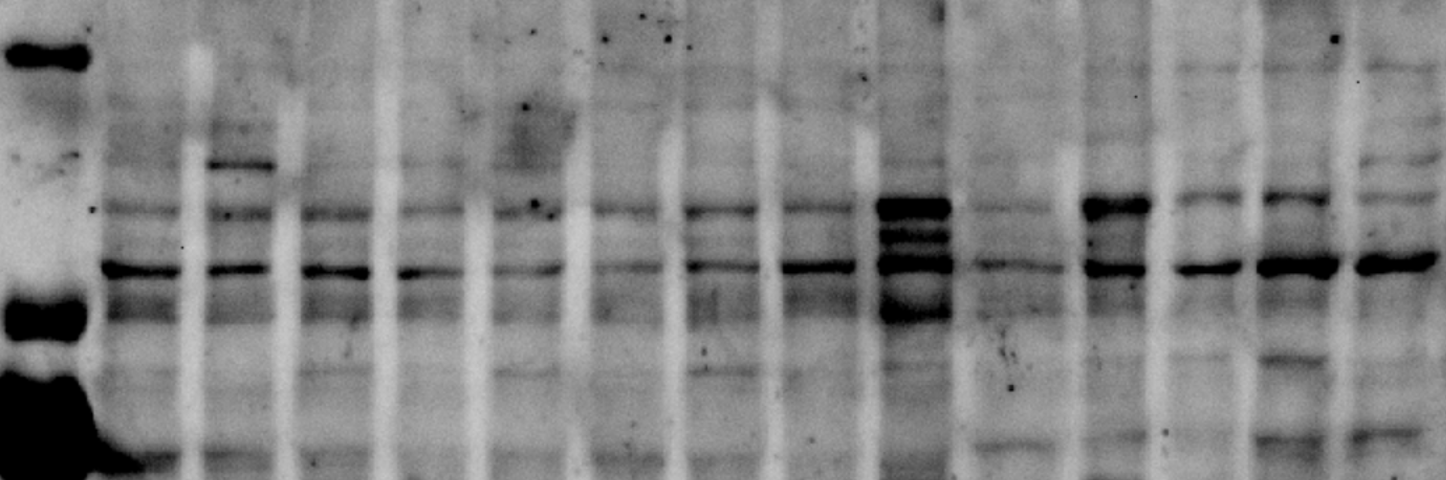

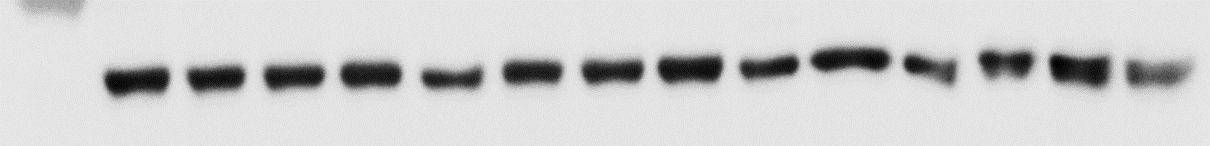


**TRPM8**

**Tubulin**

**130kDa**

**100kDa**

**70kDa**

Figure 7(c): Original uncropped blot of TRPM8 and Tubulin. The yellow box represents the cropped region used for the manuscript.


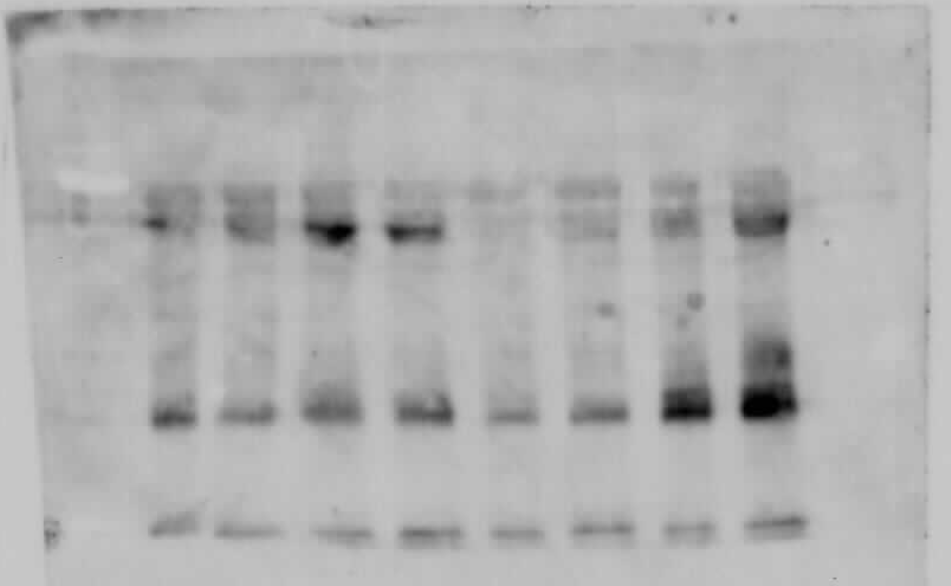


**3-nitrotyrosine**


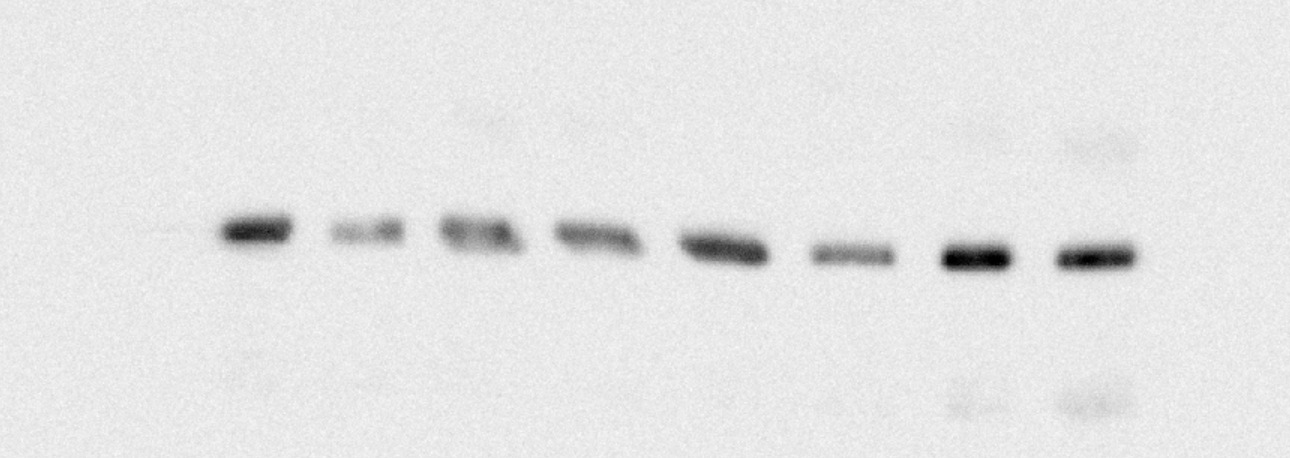


**GAPDH**

**70kDa**

Supplementary Figure 2(a): Original uncropped blot of 3-nitrotyrosine and GAPDH. The yellow box represents the cropped region used for the manuscript.


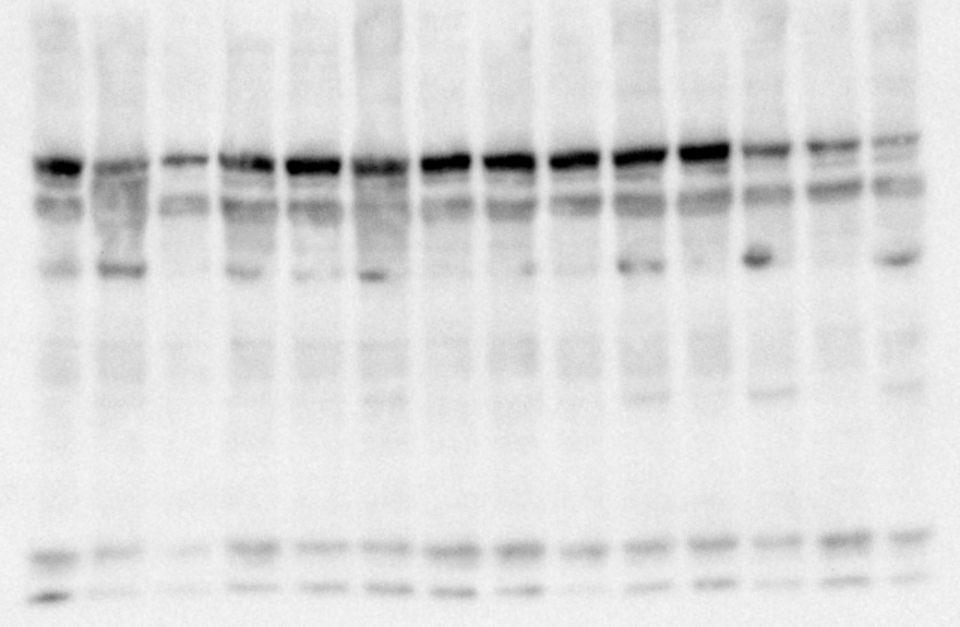


**~60kDa**

**Phospho Tyrosine hydroxylase**


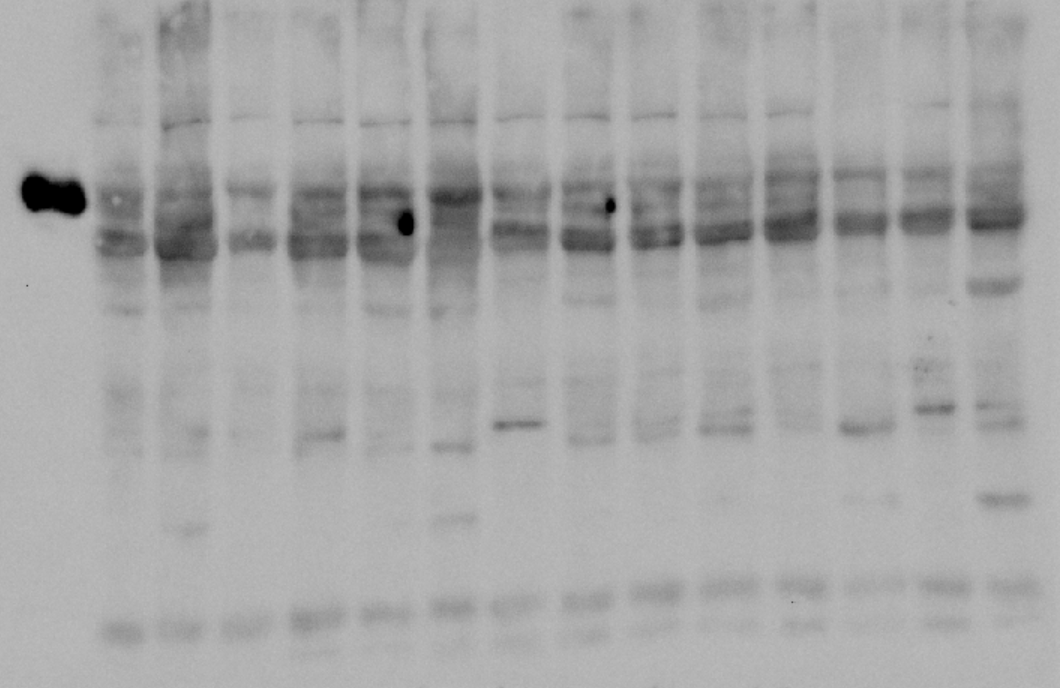


**~60kDa**

**Tyrosine hydroxylase**

Supplementary Figure 4: Original uncropped blot of phospho and total tyrosine hydroxylase. The yellow box represents the cropped region used for the manuscript.


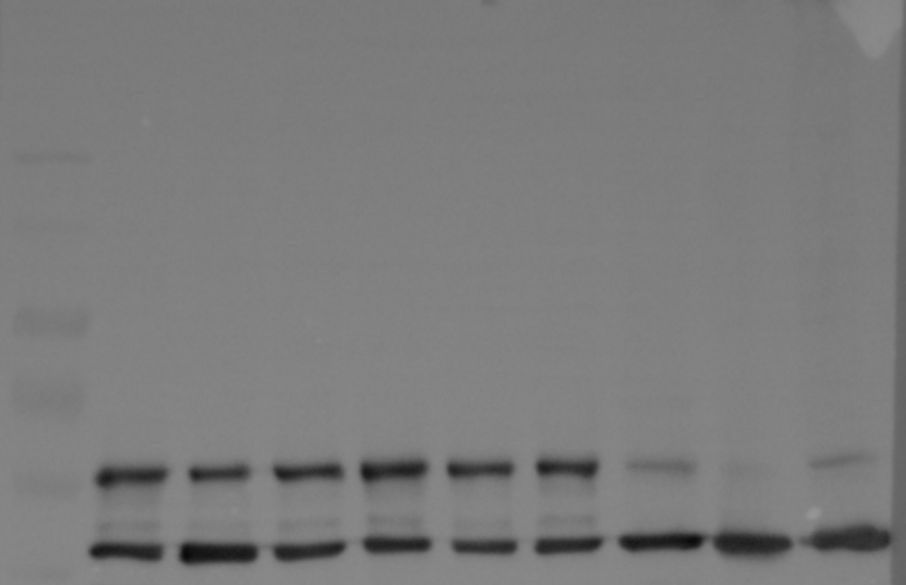


**70kDa**

**55kDa**

**40kDa**

**Tyrosine hydroxylase**

**Actin**

Supplementary Figure 6(b): Original uncropped blot of Tyrosine hydroxylase and Actin. The yellow box represents the cropped region used for the manuscript.
